# Chromosome-Level Genome Sequence of Aspergillus puulaauensis MK2, a Fungus Isolated from a Dead Hard Tick

**DOI:** 10.1128/MRA.00372-21

**Published:** 2021-09-09

**Authors:** Taiki Futagami, Kazuki Mori, Chihiro Kadooka, Hiroko Niihara, Kosuke Tashiro, Hisanori Tamaki, Tetsuya Tanaka

**Affiliations:** a United Graduate School of Agricultural Sciences, Kagoshima University, Kagoshima, Japan; b Education and Research Centre for Fermentation Studies, Faculty of Agriculture, Kagoshima University, Kagoshima, Japan; c Cell Innovator Co., Ltd., Fukuoka, Japan; d Laboratory of Infectious Diseases, Joint Faculty of Veterinary Medicine, Kagoshima University, Kagoshima, Japan; e Laboratory of Molecular Gene Technology, Faculty of Agriculture, Kyushu University, Fukuoka, Japan; University of California, Riverside

## Abstract

Aspergillus puulaauensis strain MK2 was isolated from a dead hard tick (Haemaphysalis longicornis). Here, we determined the chromosome-level genome sequence of A. puulaauensis MK2.

## ANNOUNCEMENT

During a Haemaphysalis longicornis hard tick study, we observed that the ticks were covered with fungi, resulting in subsequent tick death. H. longicornis is known as a vector of several pathogens causing illnesses, including *Theileria* spp., *Babesia* spp., *Rickettsia* spp., and severe fever with thrombocytopenia syndrome virus (1). We isolated Aspergillus puulaauensis MK2 from the dead tick. The species was identified based on the DNA sequence of the β-tubulin-encoding *benA* gene with direct colony PCR using a primer set of Bt_2_a and Bt_2_b (2). The *benA* gene exhibits 100% identity to those of A. puulaauensis NRRL 58602, which was isolated from indoor air in West Virginia, USA, and is known to produce sterigmatocystin (3, 4).

**FIG 1:**
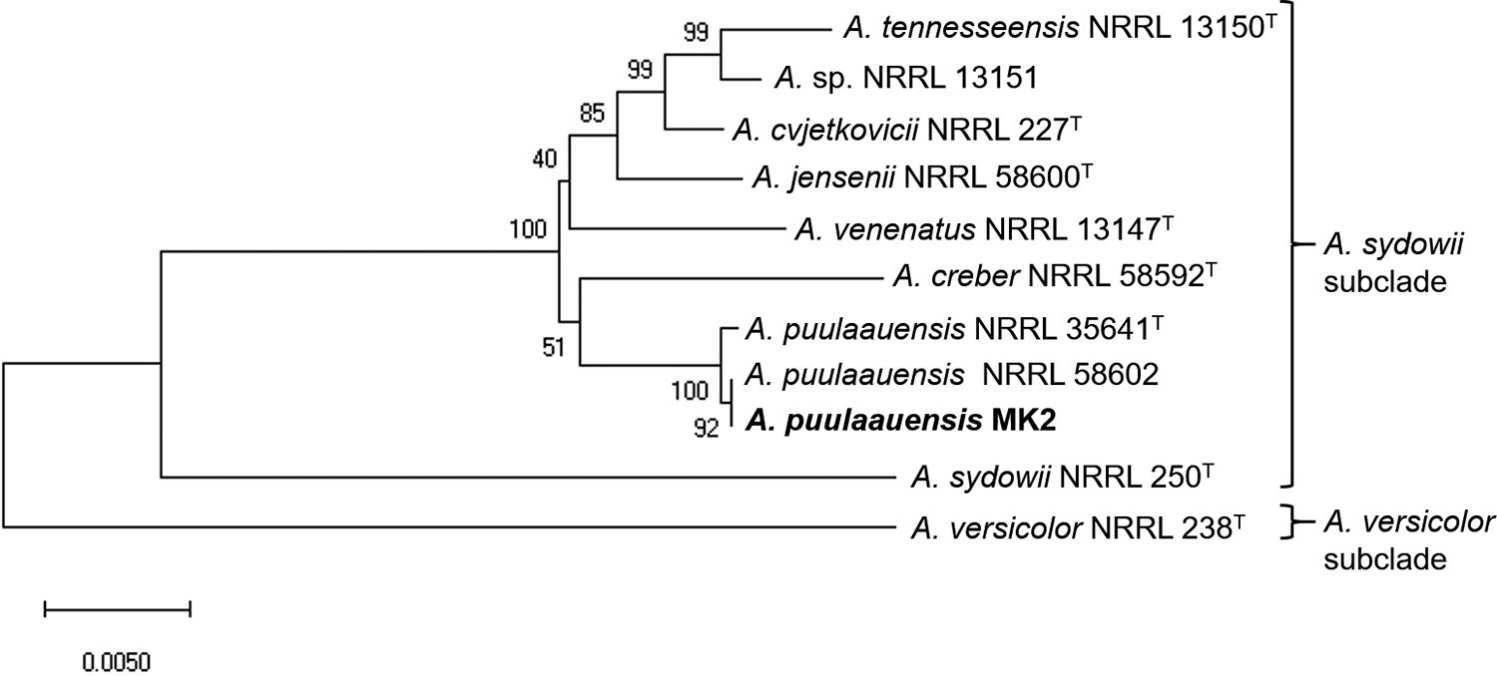
Phylogenetic analysis of Aspergillus versicolor clade strains, showing the phylogenetic position of strain MK2. The position of strain MK2 is indicated by bold type. The tree was constructed by a neighbor-joining method based on an alignment of concatenated *benA*, *caM*, *tsr1*, and *rpb2* sequences with complete gap deletion with MEGA X (34). Bootstrap values (1,000 replicates) are indicated at the branches. All of the other analytical options were set to defaults. Sequence data were obtained from the DDBJ using the following accession numbers: NRRL 13150 *benA*, JN853976.1; NRRL 13150 *caM*, JN854017.1; NRRL 13150 *tsr1*, JN853872.1; NRRL 13150 *rpb2*, JN853806.1; NRRL 13151 *benA*, JN853977.1; NRRL 13151 *caM*, JN854018.1; NRRL 13151 *tsr1*, JN853874.1; NRRL 13151 *rpb2*, JN853807.1; NRRL 227 *benA*, JN853971.1; NRRL 227 *caM*, EF652352.1; NRRL 227 *tsr1*, JN853866.1; NRRL 227 *rpb2*, EF652176.1; NRRL 58600 *benA*, JN854007.1; NRRL 58600 *caM*, JN854046.1; NRRL 58600 *tsr1*, JN853863.1; NRRL 58600 *rpb2*, JN853835.1; NRRL 13147 *benA*, JN854003.1; NRRL 13147 *caM*, JN854014.1; NRRL 13147 *tsr1*, JN853876.1; NRRL 13147 *rpb2*, JN853803.1; NRRL 58592 *benA*, JN853980.1; NRRL 58592 *caM*, JN854043.1; NRRL 58592 *tsr1*, JN853887.1; NRRL 58592 *rpb2*, JN853832.1; NRRL 35641 *benA*, JN853979.1; NRRL 35641 *caM*, JN854034.1; NRRL 35641 *tsr1*, JN853895.1; NRRL 35641 *rpb2*, JN853823.1; NRRL 58602 *benA*, JN853999.1; NRRL 58602 *caM*, JN854048.1; NRRL 58602 *tsr1*, JN853896.1; NRRL 58602 *rpb2*, JN853837.1; NRRL 250 *benA*, JN853933.1; NRRL 250 *caM*, EF652362.1; NRRL 250 *tsr1*, JN853901.1; NRRL 250 *rpb2*, EF652186.1; NRRL 238 *benA*, JN853941.1; NRRL 238 *caM*, EF652354.1; NRRL 238 *tsr1*, JN853911.1; NRRL 238 *rpb2*, EF652178.1.

To further investigate the MK2 strain, we sequenced the genome of the MK2 strain. The MK2 strain was cultured in yeast extract-peptone-dextrose medium (2% [wt/vol] glucose, 1% [wt/vol] yeast extract, and 2% [wt/vol] peptone), and then the retrieved mycelia were subjected to DNA extraction using DNAs-ici!-F (Rizo, Inc., Tsukuba, Japan) and RNA extraction using RNAiso Plus (TaKaRa Bio, Inc., Shiga, Japan) and the SV total RNA isolation system (Promega, Madison, WI). The genomic DNA of strain MK2 was sequenced to 170- and 164-fold coverage using Oxford Nanopore Technologies (ONT) MinION and Illumina NovaSeq 6000 platforms, respectively, with PCR-free workflows. ONT and Illumina sequencing libraries were prepared using the ligation 1D kit (SQK-LSK109; ONT) and the NEBNext Ultra II DNA library preparation kit (E7645; New England BioLabs [NEB]), respectively. The ONT reads and Illumina reads were used for *de novo* assembly and error correction, respectively. The ONT reads were assembled by Canu v2.0 (5) and Flye v2.8-b1674 (6). The Flye assembly was used to bridge separate contigs generated by Canu. We chose the better assembly metrics based on telomere-to-telomere genome assembly. Consequently, the genome of strain MK2 was assembled into 9 contigs, consisting of 8 chromosomes and 1 mitochondrial DNA, indicating that we successfully sequenced the nearly complete genome sequence of strain MK2. Seven chromosomes were generated only by Canu, while chromosome 1 was generated by an assembly in which 2 Canu contigs were bridged by a Flye contig. The final assembly was polished using medaka v1.0.3 (7) with ONT reads, Pilon v1.23 (8) with ONT reads, and Pilon v1.23 (8) with Illumina reads. Genome annotation of the chromosomal and mitochondrial contigs obtained was performed based on the Funannotate v1.8.1 pipeline (9) and MFannot v1.1 (10), respectively. Gene prediction was performed by using SNAP v2006-07-28 (11), AUGUSTUS v3.3.3 (12), GlimmerHMM v3.0.4 (13), and GeneMark-ES v4.61_lic (14) via the Funannotate v1.8.1 pipeline (9). For the analysis, RNA-seq reads for strain MK2 were obtained with the NovaSeq 6000 system and used for gene prediction. The sequencing library was prepared using the NEBNext Ultra directional RNA library preparation kit for Illumina (E7420; NEB). The Illumina reads were *de novo* assembled by Trinity v2.8.5 (15) and analyzed with the sequence alignment tool HISAT v2.2.0 (16). The proteins were annotated with MEROPS v12.0 (17), UniProt v2020_05 (18), MIBiG v1.4 (19), Pfam v33.1 (20), and dbCAN2 v9.0 (21) (based on the CAZy database v7/30/2020 [22]) using sequence alignment tools such as DIAMOND v2.0.6 (23) and HMMER v3.3.2 (24). Annotation was also performed using InterProScan v5.47-82.0 (25), eggNOG-mapper v1.0.3 (26) (for the eggNOG v4.5 database [27]), antiSMASH v5.1.2 (28), SignalP v4.1 (29), Phobius v1.01 (30), tRNAscan-SE v2.0.7 (31), and Barrnap v0.9 (32). The MK2 genome consists of 34,346,679 bp, with a GC content of 49.8%, and is composed of 13,627 predicted coding sequences and 162 tRNAs. Genome completeness was assessed using Benchmarking Universal Single-Copy Orthologs (BUSCO) v5.1.2 with the ascomycota_odb10 data set (33), which resulted in 99.2% complete and single-copy BUSCOs, 0.4% complete and duplicate-copy BUSCOs, 0.1% fragmented-copy BUSCOs, and 0.3% missing BUSCOs. Details regarding the replicons present are summarized in Table 1. To confirm the species-level identification of strain MK2, we performed phylogenetic analysis using *benA* and the genes encoding calmodulin (*caM*), pre-rRNA processing protein (*tsr1*), and RNA polymerase II subunit (*rpb2*) for strain MK2 and Aspergillus versicolor clade strains (2) with MEGA X software (34). The position of strain MK2 was closest to A. puulaauensis NRRL 58602 (2, 3), confirming that strain MK2 is classified as A. puulaauensis (Fig. 1).

**TABLE 1:** Replicons of Aspergillus puulaauensis strain MK2

Location^*a*^	GenBank accession no.	Size (Mb)	GC content (%)	No. of coding sequences	No. of rRNAs	No. of tRNAs
Chr. 1	AP024443.1	6.03	49.9	2,396	0	19
Chr. 2	AP024444.1	5.43	50.2	2,207	0	18
Chr. 3	AP024445.1	4.51	50.1	1,781	0	16
Chr. 4	AP024446.1	4.39	49.8	1,733	0	29
Chr. 5	AP024447.1	4.23	49.6	1,663	0	31
Chr. 6	AP024448.1	3.59	49.5	1,478	12 (69)^*b*^	7
Chr. 7	AP024449.1	3.32	49.3	1,267	0	8
Chr. 8	AP024450.1	2.82	49.3	1,089	0	7
MT	AP024451.1	0.03	24.2	14	1	27

a Chr., chromosome; MT, mitochondria.

b The number of rRNA genes is not clear, due to their highly repetitive structure. The number in parentheses indicates the estimated copy number based on the median per-base coverage.

### Data availability.

The nucleotide sequences of A. puulaauensis MK2 chromosomes and mitochondria have been deposited in DDBJ/ENA/GenBank under accession numbers AP024443 to AP024451. Raw sequence reads have been deposited in the Sequence Read Archive (SRA) under accession numbers DRX256207, DRX251723, and DRX251722.
